# Clinical, cognitive and neuropsychiatric correlates of personality in Wilson’s disease

**DOI:** 10.1007/s10072-026-09097-y

**Published:** 2026-05-25

**Authors:** Natascia De Lucia, Raffaele Lisbino, Margherita Matarazzo, Augusta Giglio, Rosa Coppola, Fabiola Di Dato, Raffaele Iorio, Gabriele Riccio, Nelson Mauro Maldonato, Antonio Terracciano, Anna De Rosa

**Affiliations:** 1 Department of Neurosciences, Reproductive and Odontostomatological Sciences, University of Naples “Federico II”, via S. Pansini 5, Naples, 80131 Italy; 2 Department of Mental and Physical Health and Preventive Medicine, University of Campania, Naples, Italy; 3 Department of Translational Medical Science, Federico II University, Naples, Italy; 4 Department of Translational Medical Science, Section of Pediatrics, University of Naples “Federico II”, Naples, Italy; 5 Department of Geriatrics, Florida State University College of Medicine, Tallahassee, FL USA

**Keywords:** Personality, Wilson’s disease, Motor disease, Neuroticism, Psychoticism

## Abstract

**Introduction:**

Wilson’s disease (WD) is an autosomal recessive disorder characterized by hepatic, neurologic, or psychiatric symptoms. Compared to other recognized clinical symptoms, the personality profile of WD patients remains under-investigated. This study compared personality traits in a sample of adults with WD and healthy controls (HC). Moreover, we assessed the cognitive and affective correlates of personality within the WD group.

**Methods:**

We enrolled 20 patients affected with genetically confirmed WD, with prevalent extrapyramidal symptoms, and 20 age and education-matched HC. Participants completed the revised Eysenck Personality Questionnaire, neuropsychological tests, and psychological questionnaires.

**Results:**

WD patients scored significantly higher on neuroticism (Cohen’s d = 1.28; Confidence Interval: 0.61–1.95) and psychoticism (Cohen’s d = 0.72; Confidence Interval: 0.08–1.36) compared to HC, but no differences were observed for extraversion. Among WD patients, neuroticism correlated with global disability, trail making test, depression, and mental health status; psychoticism correlated with memory and executive functions (all r > ± 0.5).

**Conclusion:**

We observed that WD patients are characterized by high neuroticism and psychoticism, and this personality profile is related to other clinical, cognitive, and neuropsychiatric manifestations. Identification of personality traits can enhance early detection, improve monitoring, and guide targeted management strategies for individuals with WD.

**Supplementary Information:**

The online version contains supplementary material available at 10.1007/s10072-026-09097-y.

## Introduction

Wilson’s disease (WD), first described by Kinnier Wilson [1], is an autosomal recessive disorder caused by mutations in the *ATP7B* gene, and characterized by copper accumulation in the liver, brain, kidneys, and skeletal system. WD prevalence is estimated between 1/30.000 and 1/100.000 individuals, with marked interindividual variability due to genetics, clinical presentation, and progression of neurological, hepatic, and neuropsychiatric symptoms [2]. When the disease occurs between the second and third decades of life, the most common clinical presentation is characterized by akinetic-rigid parkinsonism, postural and intention tremor, head titubation, dysarthria, ataxia or generalized dystonic syndrome [3]. Neuropsychological profiles in WD patients are characterized by impairment of attention, memory, and executive functions [4–6]. Neuropsychiatric symptoms include a wide range of emotion-related features, such as depression, anxiety or uncontrollable crying, agitation, elation, irritability, with a few cases of delusions and hallucinations [7]. For example, Akil et al. [8] retrospectively analyzed the history of psychiatric symptoms obtained from patients’ reports and medical records, and observed that the psychiatric disorders were common among WD patients. They also noted frequent “personality changes”, including irritability, aggression, and depression. Oder et al. [9] found a strong association between the neurological features and the “organic personality” syndrome, which has been characterized by affective instability, irritability, temper outbursts, and impaired social judgment. Portala et al. [10] also observed that WD patients have prominent neuropsychiatric similar to severe depressive disorders, including fatiguability, reduced sexual interest and sleep, “lack of appropriate emotion”, hostile feelings, and sadness.

However, only a few studies have examined personality traits in WD patients. Personality traits represent dimensions of individual differences in tendencies to show consistent patterns of thoughts, feelings, and actions [11–13]. It has recently been observed that specific personality traits are strongly related to movement disorders. For example, psychoticism and neuroticism has been shown to increase the risk of developing Parkinson’s disease (PD) [14, 15]. In WD, Seniów et al. [16] showed that performance on Minnesota Multiphasic Personality Inventory (MMPI) in patients with bradykinesia, tremor, rigidity, dystonia, and dysarthria, were characterized by hypochondria, chronic fatigue, and poor psychophysical well-being, compared with normal adults. Notably, this finding has already been reported by Dening and Berrios [17] that, using the Personality assessment schedule in twenty-eight WD patients, observed a strong association of the neurotic trait with dysartria, bradykinesia, and rigidity. However, the MMPI instrument is most commonly used for the assessment of anxiety, depressive and other psychopathological symptoms rather than personality traits [18]. Other studies of WD patients have also not use standardized personality scales and relied on family member reports [8] or medical records [9]. Among the few exceptions, Portala et al. [19] used the self-report inventory Karolinska Scales of Personality (KSP), and reported a significant correlation between specific genetic mutations and personality traits in twelve treated patients with WD, with Trp779Stop and Thr977Met mutations showing high scores on psychopathy scales of the KSP.

To advance knowledge on the potential impact of WD on personality traits, this study used a well-established measure of personality, the Eysenck Personality Questionnaire (EPQ) to compare adults with WD to HC. Moreover, we assessed whether personality traits covary with motor, cognitive and affective functioning in WD. Based on evidence of frequent depressive symptoms and other negative emotions in WD, we expected that adults with WD would score higher on neuroticism. We also hypothesized that neuroticism would be associated with more neuropsychiatric and poorer performance on executive tests. These findings would support the idea for a strong relationship of personality and cerebral functioning in pathological conditions.

## Methods

### Participants

Participants were recruited from outpatient population attending the neurology clinic. Data were collected between December 2023 and June 2024. To be eligible for participation in this study, patients had to meet a clinical diagnosis of WD according to the European Association for Study of Liver, confirmed by the genetic test. Twenty consecutive WD patients (9 F and 11 M; mean age: 36.6 years, SD: 12.1; mean education: 12.3 years, SD: 4.5), were included in the present study; all the participants were White adults from a medium-high socioeconomic status. All participants were assessed using the Global Assessment Scale (GAS) for WD, which provides a measure of the global disability and neurological dysfunction (GAS-N). Twenty healthy controls (HC; 9 men), comparable for age (*t* =-0.163, *p*=.45) and education level (*t* = 1.219, *p*=.21), and no previous history of neurological disease, were recruited from volunteer associations. Written informed consent was obtained from all the participants, according to the Declaration of Helsinki and the local Ethics Committee approval. References for all the tests and the scales adopted in the present study are enlisted in the Appendix A (Supplemental material).

### Personality traits assessment

The personality traits were assessed using the Italian version of the revised Eysenck Personality Questionnaire (EPQ-r). The EPQ-r is a self-report questionnaire with a true/false response format measuring the following three personality dimensions: neuroticism (emotional instability and anxiety), extraversion (sociability and liveliness), and psychoticism (aggressiveness, coldness, and egocentricity). Reliability coefficients range between 0.76 and 0.90. High scores indicate a tendency toward the personality trait represented by each subscale.

### Neuropsychological assessment

The neuropsychological assessment included the Italian version of the following tests: the Montreal Cognitive Assessment (MOCA) for the general cognitive functioning; the immediate and delayed recall of the 15-Word learning test for verbal memory; the Frontal Assessment Battery (FAB) for executive functioning; the Clock drawing test for visuo-spatial skills; the Trail making test for attentional skills, visuo-motor planning, sustained attention and working memory; the Stroop-colour word test for monitoring and inhibition of irrelevant responses.

### Neuropsychiatric assessment

The neuropsychiatric assessment included the following scales: the Beck Depression Inventory – II (BDI-II) for the presence and severity of cognitive and somatic-affective components related to depressive disorder; the Apathy Evaluation Scale (AES) to identify clinically significant apathy; the Symptom Checklist-90 (SCL-90) for the presence and severity of neuropsychiatric symptoms including somatization, obsessive-compulsive, interpersonal sensitivity, depression, anxiety, hostility, phobic anxiety, paranoid ideation, and psychoticism.

### Statistical analyses

For descriptive purpose, we identified WD subjects with chronic liver disease without neurological signs (non-neurological variant) and patients who presented at least one among extrapyramidal symptoms (including fine motor skills, tremor or other involuntary movements, rigidity, bradykinesia/hypokinesia, hypophonia), ataxia, dysarthria, pyramidal signs, drooling, and gait disorders (neurological variant). Then, we checked the normality of data distribution by means of Shapiro-Wilk’s test. Then, univariate nonparametric Mann-Whitney U test was performed to detect any differences on demographic and clinical data, and scores on neuropsychological, personality and psychological tests between WD patients and HC. Spearman correlation coefficients between the raw scores on the variables considered in WD patients was calculated to assess the possible associations of the personality traits with clinical features, cognitive abilities and neuropsychiatric symptoms. All data analyses were performed using SPSS Statistics, version 24.0.

## Results

Among WD patients, fifteen patients (6 F and 9 M; 75%) were classified as affected by the neurological variant of WD, showing one or more among action tremor, mild dystonia, bradykinesia, muscle rigidity, osteotendinous hyperreflexia, and Babinski sign. Brain MRI showed high T2 signal in the brainstem and basal ganglia, and hyperintensity of the corticospinal tracts in ten of them. The remaining five patients (4 F and 1 M; 25%) were considered as affected by the non-neurological variant as denied any symptoms and presented normal neurological examination. Penicillamine was administered to two subjects (one with neurological variant and one with non-neurological variant), and trientine to one with neurological variant, whereas all other patients were treated with zinc salts. The measures obtained did not met the normality assumption (Shapiro-Wilk’s test, all *p*<.05). Our results are shown in Table 1, and showed that WD patients scored significantly higher on EPQ-r neuroticism (U = 73.50, z= -3.45, *p*<.001) and psychoticism (U = 120.00, z= -2.19, *p*=.02) compared with HC, but there were no significant differences on extraversion (U = 198.00, z=-0.043, *p*=.96). WD patients also scored significantly worse on MOCA (U = 83.00, z=-3.21), Clock drawing test (U = 122.50, z=-2.385), TMT-part A (U = 62.50, z=-3.730), TMT-part B (U = 106.00, z= -2.548), and Stroop-colour word test – time (U = 63.50, z=-3.703) compared with HC. Notably, the average MOCA score of 24 in the WD group suggest a mild level of cognitive impairment, despite the relatively young age of this sample. Moreover, WD patients scored significantly higher on BDI-II (U = 122.00, z= -2.13), AES (U = 126.00, z= -2.09), and SCL-90 (U = 39.00, z= -4.38), compared to HC. No significant differences were observed on 15-Word learning test, and FAB or Stroop-color word test - errors scores (see Table 1).

**Table 1:** Mean and standard deviation (SD) on demographic, clinical, neuropsychological, personality and psychological data in Wilson’s Disease (WD), and Healthy Control (HC)

	WD (*N* = 20)	HC (*N* = 20)	
	Mean (SD)	Mean (SD)	*p*
Demographic data			
Age	36.75 (12.48)	38.90 (10.13)	0.34
Education	13.00 (4.37)	14.25 (2.59)	0.53
*Clinical data*			
Onset disease	14.4 (15.3)	-	-
Disease duration	21.9 (9.0)	-	-
GAS - total score	7.0 (5.9)	-	-
GAS - N	5.4 (4.6)	-	-
*Neuropsychological data*			
Montreal Cognitive Assessment	24.00 (3.418)	26.80 (1.32)	**0.001**
15-Word learning test – immediate recall	43.30 (13.985)	51.45 (9.93)	0.06
15-Word learning test – delayed recall	9.40 (4.321)	11.75 (1.71)	0.15
Clock drawing test	8.425 (2.4777)	9.87 (0.22)	**0.01**
TMT – part A	49.80 (22.348)	29.60 (5.57)	**< 0.001**
TMT – part B	162.00 (116.753)	78.70 (14.85)	**0.01**
Frontal Assessment Battery	16.20 (2.397)	17.10 (1.51)	0.16
Stroop-colour word test – time	24.063 (8.5069)	14.90 (3.37)	**< 0.001**
Stroop-colour word test – errors	0.95 (1.276)	0.85 (0.93)	0.96
*Personality trait data*			
EPQ-r neuroticism	15.25 (4.51)	10.30 (2.20)	**< 0.001**
EPQ-r psychoticism	10.20 (3.62)	7.05 (4.19)	**0.02**
EPQ-r extraversion	10.35 (2.39)	10.00 (2.65)	0.96
*Psychological data*			
BDI-II	10.40 (6.770)	6.35 (1.30)	**0.03**
AES	38.60 (5.633)	20.00 (2.75)	**< 0.001**
SCL-90	72.60 (35.223)	18.95 (10.79)	**< 0.001**

GAS (Global Assessment Scale for Wilson’s Disease); GAS-N (Global Assessment Scale for Wilson’s Disease – Neurological score); TMT (Trail Making Test); EPQ-r (Eysenck Personality Questionnaire-revised); BDI-II (Beck Depression Inventory II); AES (Apathy Evaluation Scale); SCL-90 (Symptom Checklist-90); in bold the significant values

Correlation analyses in WD group showed that the scores on EPQ-r neuroticism were positively correlated with GAS total score, TMT – part A, BDI-II, and SCL-90; the scores on EPQ-r psychoticism were negatively correlated with 15-Word learning test – immediate and delayed recall, and positively with TMT – part A and part B (see Table 2).

**Table 2:** Sperman correlation coefficients between demographic and clinical data, and neuropsychological and psychological test scores with neuroticism, and psychoticism subtests of the EPQ-r in WD patients

	EPQ-*r* *N*	EPQ-*r* *P*
*Demographic data*		
Age	0.192	0.125
Education	− 0.375	− 0.388
*Clinical data*		
Onset disease	− 0.065	0.254
Disease duration	0.178	− 0.138
GAS - total score	**0.558** ^*^	0.314
GAS - N	0.141	0.062
*Neuropsychological data*		
Montreal Cognitive Assessment	− 0.357	− 0.411
15-Word learning test – immediate recall	− 0.178	**− 0.642** ^**^
15-Word learning test – delayed recall	− 0.004	**− 0.539** ^*^
Clock drawing test	− 0.274	− 0.189
TMT – part A	**0.467** ^*^	**0.449** ^*^
TMT – part B	0.367	**0.526** ^*^
Frontal Assessment Battery	− 0.257	0.036
Stroop-color word test – time	0.441	0.102
Stroop-color word test – errors	0.082	− 0.007
*Psychological data*		
BDI-II	.592^**^	0.151
AES	0.307	0.000
SCL-90	**0.645** ^**^	0.248

GAS (Global Assessment Scale for Wilson’s Disease); GAS-N (Global Assessment Scale for Wilson’s Disease – Neurological score); TMT (Trail Making Test); EPQ-r (Eysenck Personality Questionnaire-revised); BDI-II (Beck Depression Inventory II); AES (Apathy Evaluation Scale); SCL-90 (Symptom Checklist-90); in bold the significant values. *Correlation significant at *p*<.05; **correlation significant at *p*<.01

## Discussion

This study compared the personality traits of adults with WD to those of healthy controls, and examined the neuropsychological correlates of personality in these WD patients. Neuropsychological exam confirmed the impairment of executive functions in the patient group, as previously described [4, 5] and neuropsychiatric assessment showed that WD patients have more depression and apathy, and higher psychological discomfort and neuropsychiatric symptomatology. Of central interest in the current study, by adopting a theoretical-based instrument to assess the personality, we observed that our WD patients were characterized by high levels of neuroticism and psychoticism compared to HC, and there were no significant differences on extraversion. The higher neuroticism suggests that WD subjects manifest increased tendency of emotional reactivity, anxiety, and moodiness. The finding for extraversion suggests an average tendency to socialize with other people. And the higher psychoticism suggests a higher predisposition for aggression, impulsivity, non-conformity, and a lack of empathy. Consistently, affective disorders of neurotic nature, including apprehension and sensitive temperament with extreme over-reaction to events, have been conventionally described among people suffering from copper toxicity syndrome [20]. Moreover, the high score on neuroticism has been reported also in other neurological conditions, including stroke, Alzheimer, vascular dementia, mild cognitive impairment, PD and traumatic brain injury [12–14, 21–23]. In a recent study analyzing data from six large longitudinal samples of adults it has been found that higher neuroticism was associated with higher risk of incident stroke [22]. Notably, prospective studies indicate that mid-life neuroticism is a risk factor for brain-related diseases (e.g., 4, 22]. However, large increases in neuroticism have been observed with the development of the disease, such as stroke and dementia [24].

The finding of high scores on psychoticism is similar to previous studies employing structured tools to assess the personality traits. Those studies reported that WD, compared to healthy controls, scored higher especially in aggressiveness-hostility [4], bizarre sensory experiences and general health concerns traits [16]. These outcomes would be consistent with the idea that patients with WD may have personality traits related to fronto-basal deficits [16].

Due to pathogenic background, the personality differences observed in WD are likely due to the accumulation of copper and its impact on the brain function. Indeed, in WD subjects a high level of copper has been found in frontal-subcortical brain regions, such as thalamus or globus pallidus, usually involved in mood regulation and emotional control [1].

In our cohort, the neuroticism and psychoticism traits of personality correlated with neuropsychological and psychological features. These findings could suggest that specific personality traits may individually associate with clinical, neuropsychiatric and cognitive features in these patients. Furthermoore, we may hypothesize that the patients suffering from WD may show, in addition to high level of negative emotionality and vulnerability to stress, broad antisocial tendencies including coldness, egocentrism, hostility, suspiciousness, aggressiveness, risk-taking, impulsiveness, irresponsibility, manipulation, sensation-seeking, and thoughtlessness. Thus, the finding that WD patients are characterized by a psychoticism personality trait could contribute to understand why the psychiatric disorders are more frequently associated with neurological symptoms in WD patients [9, 24]. Moreover, we also found that psychoticism personality trait in WD patients positively associated with frontal dysfunctions and memory deficits, confirming the idea for a crucial role of the frontal brain circuits in the expression of psychiatric disorders and psychological distress symptomatology.

The finding of executive dysfunctions in WD compared to HC has been widely described and attributed to copper accumulation in the basal ganglia [1]. The association between executive dysfunctions and basal ganglia dopaminergic dysregulation has also been confirmed in several neurological conditions [25–27]. The impairment of visuospatial skills, cognitive flexibility and ability to inhibit cognitive interference may be due to cortical and white matter microstructural abnormalities, and a more ubiquitous accumulation of copper beyond the basal ganglia.

Beyond the dopamine dysregulation in the basal ganglia, various molecular mechanisms have been hypothesized to underlie the psychiatric symptoms pathogenesis, such as free copper-induced central nervous system toxicity, oxidative stress, mitochondrial dysfunction, mitophagy, and neurotransmitters and trophic factors changes [28]. Neuroticism has been related to mitochondrial dysfunction [29, 30] and psychoticism seems to be linked to dopamine, glutamate, acetylcholine, and low levels of monoamine oxidase. Copper acts as a cofactor for some enzymes involved in the biosynthesis and reuptake of neurotransmitters, such as dopamine, serotonin, dopamine, and noradrenaline [28]. Furthermore, copper modulates glutamatergic synaptic transmission, GABA-A receptors and NMDA receptors [28]. Neurofunctional studies are warranted in order to clarify the neuronal correlates underlying the relationship between personality traits and WD. Finally, the diagnosis itself, the symptoms and the drugs could represent stress factors leading to psychological changes.

Some weakness needs to be considered. The relatively small sample size may have resulted in a lower subject-to-variable ratio and less generalizability of the results. Moreover, the sample size may have influenced the statistical power of the study and increased the risk of Type I error. In addition, effect size estimates, including the observed correlations, may be less stable in small samples and therefore potentially inflated. Therefore, although the reported results appear strong and reliable, they should be interpreted with caution. However, the limited sample size is due, at least in part, to the rarity of the condition under investigation, which constrains patient recruitment. Overall, these results should be considered exploratory and preliminary, and require confirmation in larger, preferably multicenter, studies.

Furthermore, the cross-sectional methodology limits our ability to examine whether the observed neuropsychiatric differences were pre-existing to WD or developed due to WD. However, WD has a genetic basis and the observed large differences in personality are most likely explained by the effect of WD on the brain. For the potential theoretical and clinical implications, future longitudinal research should examine the effect of treatment on the personality profile and how personality changes with the progression of the disease. Indeed, a crucial question is whether the changes in personality can be reversed by controlling the level of copper. Another limitation is that although the EPQ-r is a valid and reliable measure of personality, it does not assess all five major personality traits [31].

In conclusion, the present study observed that our WD patients had a higher level of neuroticism and psychoticism personality traits compared to HC, that appeared to be specifically associated with neuropsychological or neuropsychiatric features.

## Supplementary Information

Below is the link to the electronic supplementary material.

Supplementary Material 1 (DOCX 14.5 KB)
