# Supplementary material for: Clinical, cognitive and neuropsychiatric correlates of personality in Wilson’s disease
Source: Neurol Sci. 2026 May 25;47(6):516. doi: 10.1007/s10072-026-09097-y (PMC13199194; doi:10.1007/s10072-026-09097-y)
Supplement: Supplementary file 1 — Supplementary Material 1 (DOCX 14.5 KB) [file 10072_2026_9097_MOESM1_ESM.docx]

**Appendix A (Supplemental material)**

Aggarwal J, Aggarwal N, Nagral A et al (2009) A novel global assessment scale for Wilson’s disease (GAS for WD). Mov Disord 24:509–518. https://doi.org/10.1002/mds.22393

Allen NB (2002) Cognitive therapy of depression. Aust N Z J Psychiatry 36:275–278. https://doi.org/10.1046/j.1440-1614.2002.01013.x

Appollonio I, Leone M, Isella V et al (2005) The Frontal Assessment Battery (FAB): Normative values in an Italian population sample. Neurol Sci 26:108–116. https://doi.org/10.1007/s10072-005-0443-4

Caffarra P, Vezzadini G, Dieci F et al (2002) A short version of the Stroop test: Normative data in an Italian population sample. Nuova Riv Neurol 12:111–115

Caltagirone C, Gainotti G, Carlesimo GA et al (1995) Batteria per la valutazione del deterioramento mentale (parte I). Arch Psicol Neurol Psichiatria 55:461–470

Conti S, Bonazzi S, Laiacona M et al (2015) Montreal Cognitive Assessment (MoCA)-Italian version: Regression based norms and equivalent scores. Neurol Sci 36:209–214. https://doi.org/10.1007/s10072-014-1921-3

Dazzi C, Pedrabissi L, Santinello M (2004) Adattamento italiano delle Scale di Personalità Eysenck per adulti. Organizzazioni Speciali, Firenze

Derogatis LR, Lazarus L (1994) SCL-90-R, Brief Symptom Inventory, and matching clinical rating scales. In: Maruish ME (ed) The use of psychological testing for treatment planning and outcomes assessment, 2nd edn. Lawrence Erlbaum, Hillsdale, pp 217–248

Giovagnoli AR, Del Pesce M, Mascheroni S et al (1996) Trail making test: Normative values from 287 normal adult controls. Ital J Neurol Sci 17:305–309. https://doi.org/10.1007/BF01997792

Marin RS, Biedrzycki RC, Firinciogullari S (1991) Reliability and validity of the Apathy Evaluation Scale. Psychiatry Res 38:143–162. https://doi.org/10.1016/0165-1781(91)90040-V

Mondini S, Mapelli D, Vestri A et al (2003) Esame neuropsicologico breve. Cortina Editore, Milano
